# Multi-Omics Analyses Reveal the Mechanisms of Early Stage Kidney Toxicity by Diquat

**DOI:** 10.3390/toxics11020184

**Published:** 2023-02-16

**Authors:** Huazhong Zhang, Jinsong Zhang, Jinquan Li, Zhengsheng Mao, Jian Qian, Cheng Zong, Hao Sun, Beilei Yuan

**Affiliations:** 1 Department of Emergency, The First Affiliated Hospital of Nanjing Medical University, Nanjing 210029, China; 2 Institute of Poisoning, Nanjing Medical University, Nanjing 211100, China; 3 Department of Urology, The First Affiliated Hospital of Nanjing Medical University, Nanjing 210029, China; 4 College of Safety Science and Engineering, Nanjing Tech University, Nanjing 211816, China

**Keywords:** diquat, kidney injury, multi-omics, fatty acid metabolism, PPAR signaling pathway

## Abstract

Diquat (DQ), a widely used bipyridyl herbicide, is associated with significantly higher rates of kidney injuries compared to other pesticides. However, the underlying molecular mechanisms are largely unknown. In this study, we identified the molecular changes in the early stage of DQ-induced kidney damage in a mouse model through transcriptomic, proteomic and metabolomic analyses. We identified 869 genes, 351 proteins and 96 metabolites that were differentially expressed in the DQ-treated mice relative to the control mice (*p* < 0.05), and showed significant enrichment in the PPAR signaling pathway and fatty acid metabolism. Hmgcs2, Cyp4a10, Cyp4a14 and Lpl were identified as the major proteins/genes associated with DQ-induced kidney damage. In addition, eicosapentaenoic acid, linoleic acid, palmitic acid and (R)-3-hydroxybutyric acid were the major metabolites related to DQ-induced kidney injury. Overall, the multi-omics analysis showed that DQ-induced kidney damage is associated with dysregulation of the PPAR signaling pathway, and an aberrant increase in Hmgcs2 expression and 3-hydroxybutyric acid levels. Our findings provide new insights into the molecular basis of DQ-induced early kidney damage.

## 1. Introduction

Pesticides are the leading cause of poisoning-related accidental deaths in China. Following the discontinuation of paraquat, diquat (DQ) has become the preferred bipyridyl herbicide. However, cases of DQ poisoning have continued to increase in recent years, and the predominant route of exposure is the gastrointestinal tract [1]. The kidney is the main excretory organ as well as the primary target of DQ, and the toxic effects of the latter mainly involve the renal tubules, eventually leading to acute kidney injury (AKI) [2]. The incidence of AKI in patients with DQ poisoning is 73.3%, which is significantly higher compared to that caused by paraquat or other pesticides.

Previous studies have shown that DQ is selectively toxic to the kidneys, and has a similar chemical structure to that of the highly nephrotoxic orellanine [2]. Renal tubular dysfunction is the initial manifestation of DQ toxicity [3], and obvious renal tubular epithelial cell damage has been observed during autopsy [4]. The offspring of DQ-intoxicated rats exhibit renal duct damage. Furthermore, the prognosis of patients with DQ poisoning is closely related to AKI, which is usually reversible in the early stage. However, given the narrow time window for treatment, the incidence of endpoint events (death or uremia) exceeds 30%. Therefore, early detection and prevention of AKI are crucial in cases of DQ poisoning [5,6,7]. 

The clinical diagnosis of AKI is currently based on elevated blood creatinine (Scr) and blood urea nitrogen (BUN), along with low urine output [7]. However, the rise in Scr and BUN is increased when renal function has already declined by nearly 50%, while the urine output is susceptible to multiple factors such as diuretics and blood volume. Moreover, Scr and BUN are easily cleared by continuous renal replacement therapy (CRRT) and the urine volume varies with the ultrafiltration volume of CRRT. Thus, none of these indicators can accurately reflect the changes in renal function during CRRT [8]. Therefore, it is unclear whether using high Scr and oliguria as the clinical criteria for the initiation of CRRT delays the clearance of nephrotoxic substances such as DQ, and whether hemoperfusion (HP) combined with early CRRT improves prognosis [2,8]. Therefore, it is crucial to identify novel biomarkers and effector molecules for early detection and progression of kidney injury, and to guide hemodialysis treatment.

In this study, we used integrated metabolomics, transcriptomics and proteomics to explore the molecular mechanisms underlying DQ-induced nephrotoxicity at the very early stage. Based on multi-omics analyses, we found that DQ induced aberrant gene expression at the mRNA, protein, and metabolite levels. Our findings provide novel insights into DQ-induced kidney injury and identify novel biomarkers.

## 2. Materials and Methods

### 2.1. Animals and Chemical Reagents Treatments

Male C57BL/6 J mice aged 28 weeks and weighing 25–30 g were bought from Nanjing Medical University (NYD-L-2020082601). The mice were kept in a specialized pathogen-free environment (22–26 °C, 40%–60% humidity, and 12 h light/dark cycles) with food and water provided ad libitum. The feed used in this experiment meets the national standard. The feed mainly contains energy, protein, fat, amino acid, minerals, etc. All mice were given the same food. The mice were randomly divided into the control, low-dose DQ (200 mg/kg) and high-dose DQ (350 mg/kg) groups after one week of acclimatization (*N* = 30 per group). DQ and saline (control) were administered via the intragastric route. The mice were euthanized on days 1, 3 and 7 after induction, and kidney tissue samples were collected from 10 mice of each group. Ten kidney samples were used for metabolomics analysis, three were used for proteomics analysis, and three for transcriptomic analysis. Diquat (DQ) was purchased from Aladdin (D101258-100 mg).

### 2.2. Histopathologic Examination

The kidney tissues were fixed in 4% paraformaldehyde for 24 h, dehydrated in an ethanol gradient and embedded in paraffin. The paraffin blocks were cut into 5 µm-thick slices, which were stained using hematoxylin and eosin (H&E). Instrument information: Tissue-tekvip6 automatic tissue processor (Sakura, Japan), HistoStar tissue burying machine (Thermo, US), Thermo Finesse E+ paraffin microtome (Thermo, US), Gemini AS automatic dyeing machine (Thermo, US), Olympus BX53 optical microscope (Olympus, Japan), and DP72 image analysis system (Olympus, Japan).

### 2.3. Transcriptome Analysis

RNA sequencing (RNA-seq) was performed on three biological replicates of the DQ-treated and control group kidney tissues by Biotree Biotech Co., Ltd. (Shanghai, China). Briefly, total RNA was extracted and reverse transcribed, and the double-stranded cDNA was used to construct libraries. After quality control, the libraries are pooled and sequenced on the Illumina Novaseq 6000 platform (Thermo, US). The clean reads were filtered from the raw sequencing data after checking for the sequencing error rate and the distribution of GC content. The gene expression levels were calculated as the number of fragments per kilobase of transcript per million reads (FPKM). The expression matrix of all samples was generated, and differentially expressed genes (DEGs) between the control and DQ-treated samples were screened using the edgeR program with *P*adj < 0.05 as the criterion. The DEGs were then functionally annotated by gene ontology (GO) analysis in terms of molecular functions (MF), biological processes (BP) and cellular components (CC), as well as Kyoto Encyclopedia of Genes and Genomes (KEGG) pathway enrichment analyses using the clusterProfiler (http://www.bioconductor.org/packages/release/bioc/html/clusterProfiler.html) program, (accessed on 31 December 2021). The GO terms related to molecular function, biological process and cellular component were analyzed. 

### 2.4. Proteomics Analysis

Total protein was extracted from the kidney tissues of three biological replicates from the control and DQ-treated groups, quantified and stored at −80 °C. Proteomic sequencing and analysis were conducted by Biotree Biotech Co., Ltd. (Shanghai, China). Briefly, the extracted proteins were first quantified by the BCA assay, precipitated using acetone, and then subjected to reduction, alkylation, digestion, TMT labeling, SDC cleanup, peptide desalting and high-pH pre-fractionation. For nanoLC–MS/MS analysis, 2 µg total peptides from each sample was separated and analyzed using a nano-UPLC (EASY-nLC1200) coupled to Orbitrap Exploris 480 (Thermo Fisher Scientific) with a nano-electrospray ion source. Data-dependent acquisition (DDA) was performed in profile and the positive mode with Orbitrap analyzer for 90 min. The Tandem Mass Tag (TMT) was used to identify the proteins and screen for unique peptides with *p*-Value < 0.05 (Student’s *t* test) and fold change > 1.5 as the criteria. The proteins were subjected to principal component analysis (PCA), volcano plot analysis, hierarchical clustering analysis, GO and KEGG analyses, and protein–protein interaction (PPI) network analysis. 

### 2.5. Untargeted LC–MS Metabolomics Analysis

The kidney tissue samples from the control and DQ-treated groups (10 biological replicates per group) were prepared as previously described [9]. Metabolomic sequencing and analysis were performed by Biotree Biotech Co. Ltd. (Shanghai, China). The metabolic profiles were acquired using Quadrupole-Electrostatic Field Orbitrap Mass Spectrometer (Thermo Fisher Scientific). The single peak corresponding to each metabolite was filtered, and the missing values in the original data were reproduced. The internal standard was utilized for normalization, and the outliers were filtered based on the relative standard deviation. Partial least squares discriminant analysis (PLS-DA) and unsupervised principal component analysis (PCA) were used to identify the differential metabolites between two groups, with VIP > 1 and *p* < 0.05 as the criteria. The differential metabolites were subjected to correlation analysis, KEGG pathway analysis, and hierarchical clustering.

### 2.6. Statistical Analysis

Data visualization was performed using GraphPad Prism 5. The data were expressed as the mean ± standard deviation of the mean (SD). Data were processed by GraphPad Prism 5. The mean values were statistically analyzed by unpaired t-tests and the significant differences among different groups were assessed by a non-parametric test. Differences were considered statistically significant at *p* < 0.05.

## 3. Results

### 3.1. Establishment and Validation of DQ-Treated Mouse Model 

**Figure 1:**
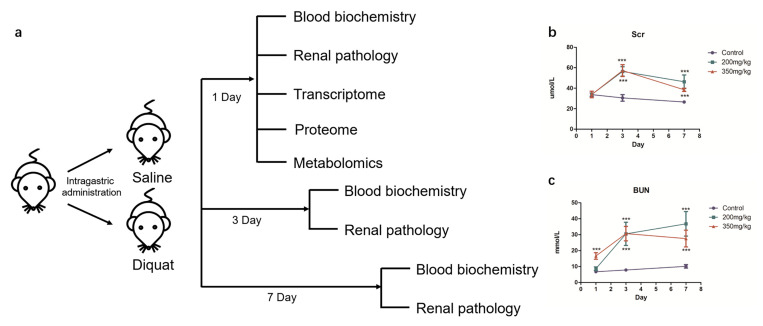
Establishment and validation of DQ-treated mice. (**a**) Outline of animal experiments. (**b**) The concentration of Scr (∗∗∗ *p* < 0.001). (**c**) The concentration of BUN (∗∗∗ *p* < 0.001).

We established a mouse model of DQ-induced kidney injury to study the early stages of AKI (Figure 1a). While DQ did not affect serum Scr levels on day 1, serum BUN levels were not affected by 200 mg/kg or 350 mg/kg DQ. The serum UREA levels were significantly higher in mice treated with 350 mg/kg DQ compared to the control group. In contrast, 200 mg/kg DQ had no significant effect on the urea level. Subsequently, both Scr and BUN continued to rise, and significant differences were observed on the 3rd and 7th days (Figure 1b,c). Furthermore, while no substantial lesions were observed in the kidney tissues of the DQ-treated mice in the first day of exposure, the renal tubules exhibited vacuolation and necrosis 3 days later (Figure S1). Based on these results, we selected the dose of 200 mg/kg to simulate the early stage DQ-induced kidney damage.

### 3.2. Transcriptomic Analysis of DQ-Treated Mice

**Figure 2:**
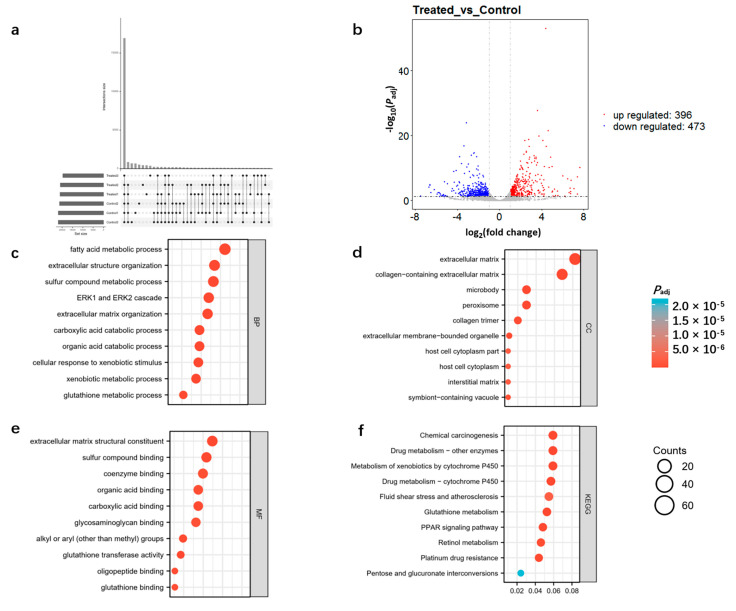
Transcriptome profiles of control and DQ-treated kidney tissues. (**a**) Summary of transcriptome datasets: 16,927 genes were expressed in all samples, *N* = 3. (**b**) The volcano plot of differentially expressed genes (DEGs) between the control and DQ-treated groups. (**c**) The enriched biological processes of DEGs. (**d**) The enriched cellular components of DEGs. (**e**) The enriched molecular function of DEGs. (**f**) The top 10 enriched Kyoto Encyclopedia of Genes pathways of DEGs and their contraction.

As shown in the UpSet graph in Figure 2a, 16,927 genes were expressed in all samples. Furthermore, 869 genes were differentially expressed in the DQ-treated samples relative to the control, of which 473 genes were downregulated and 396 genes were upregulated (Figure 2b and Table S1). The DEGs were enriched in GO terms related to fatty acid metabolism, extracellular structure organization, sulfur compound metabolism (Figure 2c), extracellular matrix, collagen-containing extracellular matrix (Figure 2d), extracellular matrix structural constituent, and sulfur compound binding (Figure 2e). Furthermore, KEGG analysis revealed that these DEGs were significantly associated with pathways of drug metabolism, drug metabolism-cytochrome P450, glutathione metabolism and retinol metabolism (Figure 2f). These results indicate that DQ might dysregulate numerous pathways in the kidneys.

### 3.3. Proteomic Analysis of DQ-Treated Mice

**Figure 3:**
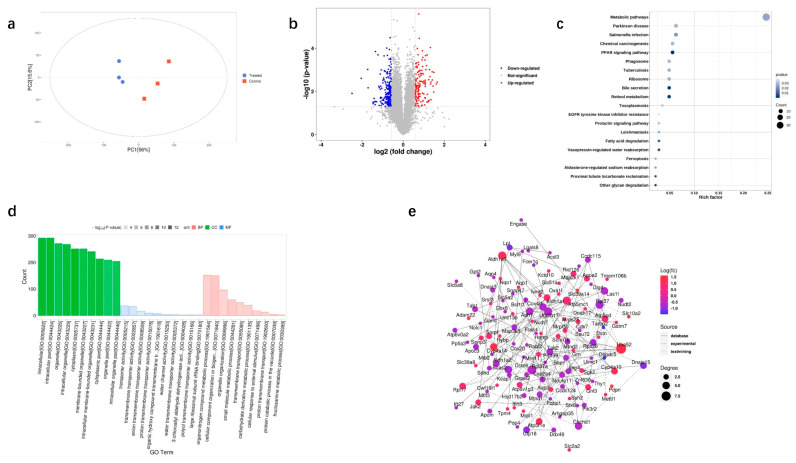
Proteomic profiles of control and DQ-treated kidney tissues. (**a**) PCA of the proteomes of DQ-treated and control kidney samples indicated two distinct clusters (*N* = 3). (**b**) Volcano plot of differentially expressed proteins (DEPs) between the control and DQ-treated groups. (**c**) Kyoto Encyclopedia of Genes and (**d**) Genomes pathways of DEPs and their contraction. (**e**) PPI network of the DEPs. The red circles represent significantly upregulated proteins, and the purple circles represent significantly downregulated proteins. The size of the circle is positively correlated with the degree of connection.

We used TMT-based quantitative proteomics analysis to identify the differentially expressed proteins (DEPs) that might be linked to DQ-induced kidney damage. PCA revealed notable differences in protein abundance between the DQ and control groups (Figure 3a). There were 351 DEPs between the two groups, of which 133 proteins were upregulated and 218 proteins were downregulated in the DQ-treated mice (Figure 3b and Table S2). The DEPs were mainly enriched in pathways associated with Parkinson’s disease, Salmonella infection, chemical carcinogenesis, PPAR signaling, phagosome, tuberculosis, ribosome, bile secretion and retinol metabolism (Figure 3c). According to the GO enrichment analysis, DEPs were primarily associated with terms such as intracellular, intracellular part, organelle, intracellular organelle, cytoplasm, membrane-bounded organelle, intracellular membrane-bounded organelle, cytoplasm part, organelle part and intracellular organelle part (Figure 3d).

Furthermore, a protein–protein interaction (PPI) network was constructed using the STRING database. As shown in the network in Figure 3e, DQ exposure altered ribonucleoprotein complex biogenesis (Bop1, Tarbp2, Imp4, Pqbp1, Pop4, Snrpf, Las1l, Mrpl1, Utp18, Ddx49, Prpf39), ncRNA processing (Bop1, Mettl1, Tarbp2, Imp4, Pop4, Las1l, Mrpl1, Utp18, Ddx49), ncRNA metabolic process (Bop1, Mettl1, Tarbp2, Imp4, Pop4, Las1l, Mrpl1, Utp18, Ddx49), response to wounding (Aqp1, Fcer1g, Pdpn, Grn, Jak2, Scnn1b, Tarbp2, Map2k1, Arhgap35), mitochondrial protein complex (Cox4i1, Grpel2, Mrps25, Chchd1, Dnajc15, Sdhd, Ndufa11, Mrpl1, Mrpl30), ribosome (Rpl37a, Uba52, Rps26, Mrps25, Chchd1, Rpl37, Mrpl1, Mrpl30, Rpl17), enzyme activator activity (Apoa2, Bcl10, Thy1, Map2k1, Dnajc15, Tab1, Cwf19l1, Arhgap35, Depdc5), organic hydroxy compound transport (Apoa2, Aqp1, Aqp3, Fcer1g, Slc10a2, Apom, Sdhd, Slc51a), fatty acid metabolic process (Adh7, Apoa2, Cyp2a4, Cyp4a10, Pdpn, Lpl, Gstm7, Acsl3), and positive regulation of cell activation (Bcl10, Fcer1g, Pdpn, Jak2, Thy1, Lgals8, Dnaja3, Hamp). In summary, DQ-induced kidney injury is likely mediated by dysregulated proteins involved in metabolism. 

### 3.4. Integrated Transcriptome and Proteome Datasets

**Figure 4:**
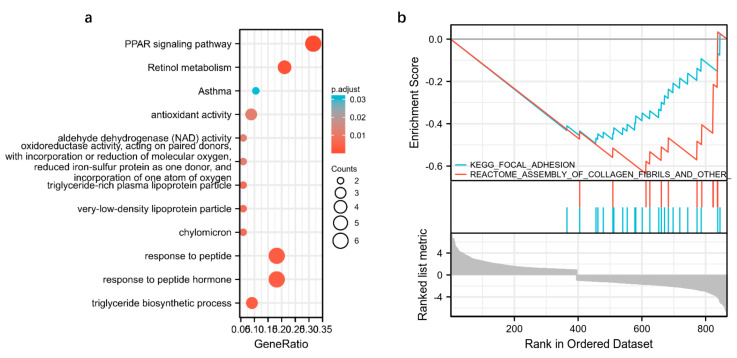
Gene set enrichment analysis (GSEA)-based analysis. (**a**) Pathways enriched in the 34 overlapping genes. (**b**) Results of GSEA.

Integration of the transcriptome and proteome datasets revealed that 34 genes were substantially altered by DQ exposure (Table S3). KEGG pathway analysis showed that these genes are significantly associated with the PPAR signaling pathway, retinol metabolism, asthma, cholesterol metabolism, fatty acid degradation, valine/leucine and isoleucine degradation, fatty acid metabolism, and kidney injury caused by DQ (Figure 4a). Furthermore, GSEA consistently demonstrated that these DEGs and DEPs were substantially enriched for metabolism-related pathways, including the drug metabolism cytochrome P450, the PPAR signaling pathway, retinol metabolism, metabolism of lipids, amino acid metabolism, glutathione metabolism and fatty acid metabolism (Figure 4b). Taken together, the aforementioned pathways are likely targeted by DQ during kidney injury.

### 3.5. Metabolomic Analysis of DQ-Treated Mice

**Figure 5:**
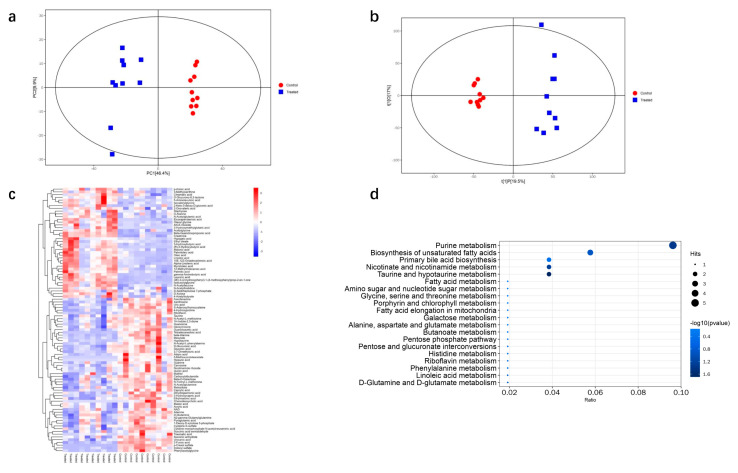
Metabolomic analysis of DQ-treated mice. (**a**) PCA of the metabolic profiles (*N* = 10). (**b**) Score plots of the OPLS-DA model classifying the DQ-treated and control groups. (**c**) Heatmap analysis of the differently expressed metabolites between the DQ-treated and control groups. The columns show the significantly upregulated (red) or downregulated metabolites (blue) in the different groups. (**d**) KEGG pathway analysis based on the differently expressed metabolites identified in the DQ-treated and control groups.

The metabolic by-products that may contribute to DQ-induced kidney injury were identified by untargeted LC–MS. The results of PCA and OPLS-DA clearly showed distinct metabolic patterns of the control and DQ-treated mice (Figure 5a,b). Overall, 96 metabolites were differentially expressed between the control and DQ-treated groups (adjusted *p* < 0.05), of which 40 were elevated and 56 were decreased in the latter (Figure 5c, Table S4). Furthermore, five of these differentially regulated metabolites are involved in purine metabolism, three in biosynthesis of unsaturated fatty acids, two in primary bile acid biosynthesis, one in fatty acid biosynthesis, and one in fatty acid metabolism (Figure 5d). To ascertain which metabolic pathways were most affected by DQ exposure, we performed KEGG pathway enrichment analysis. As shown in Figure 5d, the top 10 pathways were those related to purine metabolism, biosynthesis of unsaturated fatty acids, primary bile acid biosynthesis, nicotinate and nicotinamide metabolism, taurine and hypotaurine metabolism, fatty acid metabolism, amino sugar and nucleotide sugar metabolism, glycine, serine and threonine metabolism, porphyrin and chlorophyll metabolism, fatty acid elongation in mitochondria.

### 3.6. Integrated Transcriptomic, Proteomic and Metabolomics

**Figure 6:**
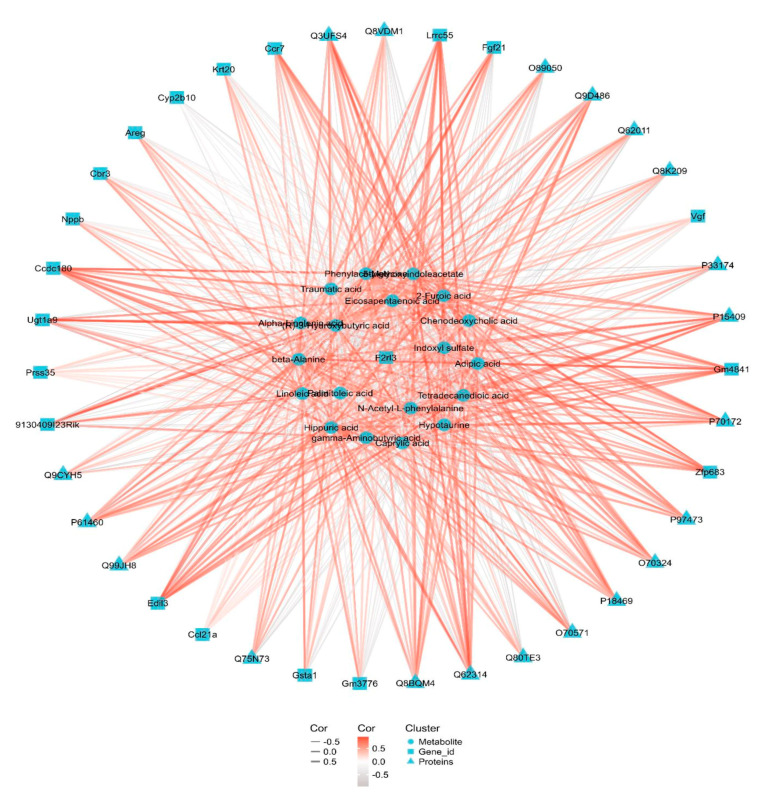
Integrative network of transcriptome, proteome and metabolome datasets.

We constructed a correlation network diagram of the metabolites, DEPs and DEGs to gain further insights into the molecular mechanisms underlying DQ-induced nephrotoxicity. As shown in Figure 6, the top 20 co-related genes were Gm3776, Ccl21a, Vgf, Gsta1, Fgf21, Krt20, Ugt1a9, Lrrc55, Areg, 9130409I23Rik, Ccdc180, Edil3, Prss35, Cbr3, Ccr7, Nppb, Cyp2b10, F2rl3, Gm4841, Zfp683. The top 20 co-related proteins were Q3UFS4, Q9D486, Q62314, O70324, O89050, Q62011, Q8K209, Q9CYH5, P61460, Q99JH8, Q80TE3, O70571, Q8BQM4, Q75N73, P97473, P15409, P33174, P70172, P18469 and Q8VDM1. The top 20 metabolites were hippuric acid, 5-methoxyindoleacetate, chenodeoxycholic acid, tetradecanedioic acid, indoxyl sulfate, (R)-3-hydroxybutyric acid, traumatic acid, gamma-aminobutyric acid, alpha-linolenic acid, adipic acid, phenylacetylglycine, hypotaurine, 3-hydroxybutyric acid, palmitoleic acid, caprylic acid, eicosapentaenoic acid, linoleic acid, 2-furoic acid, beta-alanine and N-acetyl-L-phenylalaninex. These genes, proteins and their metabolites are mostly connected to the PPAR signaling pathway and fatty acid metabolism.

## 4. Discussion

DQ is a highly nephrotoxic bipyridine herbicide that primarily targets the renal tubules and induces AKI. The molecular basis of DQ-induced kidney injury is cell death due to excessive production of reactive oxygen species (ROS) formed during lipid peroxidation [2,10]. The prognosis of DQ poisoning is highly correlated with AKI. Although AKI is reversible in its early stages, the therapeutic window is narrow. Therefore, it is crucial to identify the biomarkers and effectors of the incipient stages of AKI for early diagnosis of kidney damage. 

We identified the time window of DQ-induced kidney damage by analyzing different time points and dosages. There was no evident renal parenchymal damage, or any changes in serum Scr or BUN levels after 24 h exposure to 200 mg/kg DQ, which corresponded to the early stage of the DQ-induced kidney damage. To identify the molecular mechanisms of DQ-induced renal damage at this stage, we used an integrated multi-omics approach, which revealed that exposure to DQ significantly affects the PPAR signaling pathway and fatty acid metabolism. 

According to the integrated multi-omics data, the PPAR signaling pathway and fatty acid metabolism were associated with upregulation of Hmgcs2, Cyp4a10 and Cyp4a14, and the downregulation of Lpl mRNA and proteins in the DQ-treated kidneys. PPAR, a lipid-activated nuclear receptor, is abundantly expressed in tissues with high fatty acid metabolism, such as the kidney [11]. PPAR-deficient mice accumulate more lipids in their kidneys, which increases production of inflammatory mediators, eventually leading to kidney injury [12,13]. In addition, PPAR is also a transcription factor that controls genes involved in lipid metabolism and the mitochondrial fatty acid oxidation pathway [14], which fulfills a significant portion of the body’s energy needs [15,16]. Integrated proteomic and transcriptomic analysis revealed that the fatty acid oxidation pathway, and subsequently fatty acid metabolism, were downregulated in the DQ-treated group.

The primary rate-limiting enzyme for ketogenesis is Hmgcs2 (3-hydroxy-3-methylglutaryl-CoA synthase 2). Hmgcs2 is a key rate-regulating enzyme for ketone body formation, which is related to fatty acid metabolism and mainly exists in cell mitochondria. The HMG-CoA generated by it is converted into acetoacetic acid under the action of HMG-CoA lyase, and acetoacetic acid can be converted into hydroxybutyric acid and acetone, which are called ketone bodies. Ketogenesis of cells is an important part of fatty acid metabolism, and acetyl CoA, the product of fatty acid oxidation, is the raw material for the formation of ketosomes. Therefore, Hmgcs2 may regulate the changes in fatty acid metabolism by regulating the ketogenesis process. Upregulation of Hmgcs2 in the glomeruli of high fructose-fed rats and high fructose-treated differentiated podocytes enhanced ketone bodies level, particularly that of hydroxybutyrate (3-OHB), to block histone deacetylase (HDAC) activity [17]. Hmgcs2 is likely upregulated through the PPAR-α pathway [18]. The findings imply that enhanced renal ketogenesis due to Hmgcs2 overexpression may be significant in the pathogenesis of diabetic neuropathy DN in patients with type 2 diabetes, indicating that Hmgcs2 is a potential therapeutic target for the management of diabetic renal complications [19]. We found that Hmgcs2 gene and protein expression levels increased in the kidney tissues after DQ exposure, indicating its role in DQ-induced renal damage as well.

CYP4A (cytochrome P450, family 4, subfamily a) catalyzes the hydroxylation of medium- and long-chain fatty acids [20]. One of the pathway for fatty acid degradation is through oxidation, in which dicarboxylic acids are formed and subsequently undergo β-oxidation from the omega end. This pathway is catalyzed by CYP450 enzymes and the peroxisomal β-oxidation pathway which are regulated by PPARα [21] The mouse genome contains four Cyp4a genes: Cyp4a10, Cyp4a12a, Cyp4a12b, and Cyp4a14—all of which are localized in chromosome 4 [22]. Murine Cyp4a10 and Cyp4a14 (homologous to human CYP4A22 and CYP4A11, respectively) are highly expressed in the liver and kidneys, and are known to convert the arachidonic acid to its metabolite 20-hydroxyeicosatetraenoic acid (20-HETE), which regulates the inflammatory response through the generation of ROS [15,22]. As a result, the aberrant expression of Cyp4a10 and Cyp4a14 observed in our study may lead to fatty acid breakdown.

LPL (lipoprotein lipase) catalyzes the hydrolysis of triglyceride (TAG), which is the rate-limiting step in the lipolysis of chylomicrons and VLDL. In addition to other cell types, myocytes and adipocytes also synthesize LPL, which is then stored in the Golgi apparatus for either intracellular breakdown or secretion onto the cell surface. Patients with nephrotic syndrome often have hyperlipidemia due to the lack of LPL activators. Furthermore, the high levels of free fatty acids in the bloodstream of these patients upregulates ANGPTL4, which may inactivate LPL by either converting the active LPL dimers into inactive monomers or as a reversible non-competitive inhibitor of LPL [23]. In this study, LPL expression was downregulated in the DQ-treated kidney tissues, indicating its role in DQ-induced nephrotoxicity.

We identified eicosapentaenoic acid, linoleic acid, palmitic acid and (R)-3-hydroxybutyric acid as significant metabolites involved in DQ-related kidney injury. Eicosapentaenoic acid, linoleic acid and palmitic acid are polyunsaturated fatty acids (PUFAs), which have been linked to a number of renal disorders. One study showed that retinoic acid signaling mediates production of toxic PUFAs [24]. Increased PUFA peroxidation by ROS initiates ferroptosis, an iron-dependent form of programmed cell death. Fatty acid oxidation in the liver produced high levels of 3-hydroxybutyrate acid, which is then transferred to extrahepatic tissues including the heart, brain and muscle to be used as a fuel. As one of the ketone bodies, 3-hydroxybutyric acid can directly promotes 3-hydroxybutyrylation of some proteins and functions as an endogenous inhibitor of histone deacetylases as well as an agonist of Gpr109a [25]. β-OHB is one of the intermediate metabolites of fatty acid oxidation. In addition to being a functional vector that transfers energy from liver to peripheral tissues under starvation stress, β-OHB is also an important signaling molecule and epigenetic regulatory molecule in vivo, regulating all aspects of life function. This study showed that glomerular podocytes damage and albuminuria production caused by fructose intake showed an increase in β-OHB beginning at week 8 of modeling and continuing until week 16 of the study deadline [17]. Therefore, β-OHB is a key metabolic substance in the occurrence and development of kidney injury. Taken together, dysregulated fatty acid metabolism may induce by the nephrotoxic effects of DQ.

Overall, Hmgcs2 upregulated and subsequently may promote 3-hydroxybutyric acid levels, dysregulating the PPAR signaling pathway. Our findings offer a new insight into the mechanisms underlying DQ-induced nephrotoxicity.

## 5. Conclusions

Our study is the first to investigate the mechanism of the early stage of DQ-induced kidney injury using a multi-omics approach. Our findings lay the foundation for diagnosing and treating renal damage following DQ exposure, and offer new insights into the molecular basis of DQ-induced kidney damage.

## Data Availability

The data presented in this study are available on request from the corresponding author. The data are not publicly available due to some data are still being analyzed.

## Supplementary Materials

The following supporting information can be downloaded at: https://www.mdpi.com/article/10.3390/toxics11020184/s1, Figure S1: Representative results of pathologic staining. Scale bars represent 100 μm in 20 × images. Black arrowheads indicate renal tubules exhibited vacuolation and necrosis.; Table S1. Differential genes in control and DQ treated groups; Table S2. Differentially proteins in control and DQ treated groups; Table S3. Differential genes/proteins both in transcriptome and proteome; Table S4. Differential metabolites in control and DQ treated groups.
