# Supplementary figures and images for: Multi-Omics Analyses Reveal the Mechanisms of Early Stage Kidney Toxicity by Diquat
Source: Toxics. 2023 Feb 16;11(2):184. doi: 10.3390/toxics11020184 (PMC9966843; doi:10.3390/toxics11020184)

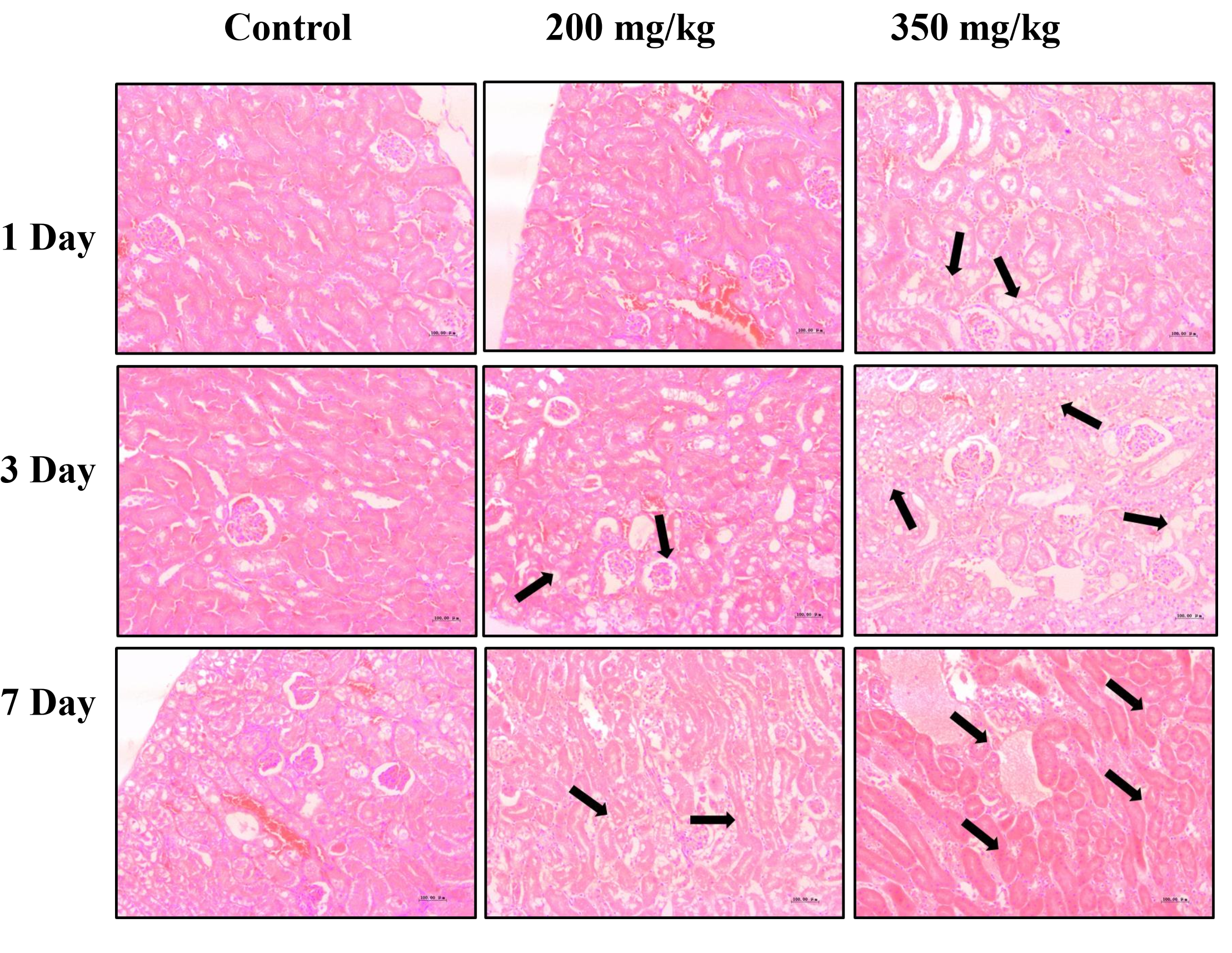

Supplement: Supplementary file 1 [file toxics-11-00184-s001.zip › Figure S1.tif]
